# Kikuchi-Fujimoto Disease in a 25-Year-Old Female: A Case Report

**DOI:** 10.7759/cureus.44007

**Published:** 2023-08-23

**Authors:** Fatima Alkhyeli, Abdollah Bahaeddin

**Affiliations:** 1 Internal Medicine, Sheikh Khalifa Medical City, Abu Dhabi, ARE

**Keywords:** tuberculous (tb) lymphadenitis, systemic lupus erythematosis, necrotizing histiocytic lymphadenitis, lymphadenopathy, kikuchi-fujimoto disease

## Abstract

Background: Kikuchi-Fujimoto disease or histiocytic necrotizing lymphadenitis is a rare benign disease that presents as cervical lymphadenopathy and fever.

Case presentation: A 25-year-old South Asian female dentist, recently married, presented to our emergency department due to two weeks of fever, sore throat, swollen neck, and cough. The patient initially presented to a private clinic and was prescribed antibiotics on two visits. On physical examination, her neck was swollen with palpable and tender right posterior and submandibular lymph nodes. Oropharyngeal examination revealed pharyngeal hyperemia without tonsillar enlargement, exudates, or mucocutaneous ulcers. Ultrasound imaging revealed enlarged neck and thoracic and abdominal lymph nodes. CBC showed leukopenia and anemia of chronic disease. B2 microglobulin, lactate dehydrogenase, and kappa light chains were elevated. Anti-ANA, anti-dsDNA, HIV polymerase chain reaction (PCR), Quantiferon-tuberculosis (TB), and rapid plasma reagin were all negative. A lymph node biopsy confirmed the diagnosis of Kikuchi-Fujimoto disease.

Conclusion: We believe this is the second case to be reported in the United Arab Emirates (UAE). Kikuchi-Fujimoto disease has a non-specific presentation that overlaps with several conditions including autoimmune, infectious, and malignant. Therefore, a thorough clinical approach and a high grade of clinical suspicion is required to rule out other possible differential diagnosis. Finally, Although Kikuchi-Fujimoto disease is mostly benign, clinicians should be careful as some patients might develop systemic lupus erythematosus (SLE), Still disease, or B cell lymphoma in the future.

## Introduction

Kikuchi-Fujimoto disease or histiocytic necrotizing lymphadenitis is a rare cause of lymphadenopathy. It usually affects females from different ethnic groups. The typical symptoms of Kikuchi-Fujimoto disease are tender lymphadenopathy, night sweats, fever, and rash [1]. This paper reports a case of Kikuchi-Fujimoto disease diagnosed in the United Arab Emirates (UAE). We report this case to supplement the literature, especially the Middle East data which might prove useful in the future.

## Case presentation

A 25-year-old South Asian female, with no significant previous medical history, presented to the emergency department with a two-week duration of high-grade fever. One week prior to presentation, the patient received azithromycin for 5 days from a private clinic. After two days, the patient returned to the private clinic due to persistent fever. She was given cefixime and ultrasound was done for the neck and abdomen for palpable cervical lymph nodes on physical examination. Ultrasound of the abdomen revealed mesenteric lymphadenopathy in the right lower quadrant measuring 24 mm into 12 mm. Ultrasound of the neck showed multiple right lateral and posterior cervical lymphadenopathy, largest lymph node measuring 20 mm into 5 mm, and a right submandibular lymph node measuring 37 mm into 12 mm.

On admission to this hospital, further history was obtained. The fever persisted for two weeks, not subsiding with paracetamol or nonsteroidal anti-inflammatory drugs. The patient was also complaining of neck swelling, sore throat, and cough. She did not notice any weight loss, night sweats, joint pain, or skin rashes. She traveled to Pakistan from August to November and to Qatar from January to March. During her travels, she denied any sick contact. She is recently married and works as a dentist. She has been sexually active since her marriage. She does not have pets and did not come into recent contact with animals.

On examination, the temperature was 39 C, heart rate 111 bpm, blood pressure 99/64 mmHg, respiratory rate 18 br/min, and oxygen saturation 98% on room air. The patient appeared fatigued and lethargic. There was no conjunctival pallor or scleral icterus. The neck was swollen on an inspection with palpable and tender posterior and submandibular lymph nodes. The lymph nodes were soft in consistency and mobile. Oropharyngeal examination revealed pharyngeal hyperemia without tonsillar enlargement, exudates, or mucocutaneous ulcers. Her chest was clear on auscultation, and her abdomen was non-tender with a palpable mass in the right lower quadrant. There was no skin rash. Her WBC count was 2.5x10^9/L, hemoglobin was 10.4, CRP was 52 mg/l, and the respiratory panel was positive for influenza B. Chest x-ray showed normal findings. Diagnostic tests were performed.

Differential diagnosis

Our patient’s presentation encompasses several diagnoses. Her occupation as a dentist, recent history of marriage, and recent onset of sexual activity can increase the likelihood of an underlying infectious cause. However, considering inflammatory and malignant causes would be just as pivotal in reaching a final diagnosis.

Tuberculous Lymphadenitis

Cervical tuberculous lymphadenitis commonly known as scrofula occurs due to reactivation of disease in a site that was seeded by hematogenous spread of a primary tuberculosis (TB) infection. It usually presents as a unilateral firm, discrete mass, or matted nodes fixed to surrounding structures, and the overlying skin may be indurated. The findings in our patient differ from the common presentation of cervical tuberculous lymphadenitis, in which the lymph nodes in our examination were tender on palpation, there were no skin changes or induration, and they were not fixed or matted. Furthermore, systemic manifestations in TB lymphadenitis such as fever only occur in 20-50% of patients [2]. In addition, there was no previous history of TB or significant illness in the patient’s history. However, primary TB can present with a mild self-resolving respiratory illness that usually does not prompt medical assistance. Although the clinical characteristics of the lymph nodes are not similar to scrofula, due to the unilaterality, her recent travel history, and occupation, TB infection should be ruled out in this patient.

Human Immunodeficiency Virus

A variety of symptoms and signs can be seen in acute symptomatic HIV infection. The most common findings are fever, lymphadenopathy, sore throat, rash, myalgia/arthralgia, diarrhea, weight loss, and headache. Oropharyngeal findings include pharyngeal edema and hyperemia, usually without tonsillar enlargement or exudate, and painful mucocutaneous ulceration. Most of the findings in acute symptomatic HIV infection are not specific and diagnosis is often missed without a high degree of suspicion. Therefore, due to the presence of non-specific findings in our patient including fever, lymphadenopathy, sore throat, and recent onset of sexual activity, we have opted to include HIV as a differential diagnosis and rule it out using laboratory tests.

Secondary Syphilis

Patients with secondary syphilis present with a variety of non-specific constitutional symptoms including fever, headache, malaise, anorexia, sore throat, myalgia, and weight loss. They can also present with lymph node enlargement in the posterior cervical, axillary, inguinal, and femoral regions and epitrochlear nodes which is suggestive of the diagnosis. The lymph nodes are tender, firm, and rubbery in consistency. They present with macular or papular eruption involving the trunk and extremities including the palms and the soles of the foot; however, this rash commonly goes unnoticed by the patient due to its inconspicuous appearance. Our patient does not have a history of chancre suggesting a primary syphilis infection. However, patients with secondary syphilis may not have a history of a preceding chancre since the primary infection may have been asymptomatic or gone unnoticed. Therefore, despite the absence of maculopapular rash in our patient or the presence of a previous history of a chancre, secondary syphilis is a differential diagnosis in our patient due to the variety of its presentations, non-specific symptoms and findings, and recent onset of sexual activity.

Systemic Lupus Erythematosus (SLE)

SLE is a chronic inflammatory autoimmune disease of an unknown etiology that affects any organ of the body and presents with a variety of manifestations. Hematological manifestations in SLE include leukopenia, anemia, thrombocytopenia, pancytopenia, lymphadenopathy, and splenomegaly. The nodes are typically soft, non-tender, discrete, varying in size, and usually detected in the cervical, axillary, and inguinal areas. SLE is a potential diagnosis in this patient due to the non-specific presentation of this condition and the presence of hematological symptoms including bicytopenia and cervical lymphadenopathy.

Sarcoidosis

Sarcoidosis is a multisystem granulomatous disease of an unknown etiology. Lymphadenopathy is one of the extrapulmonary manifestations of sarcoidosis which usually presents as hilar and\or paratracheal mediastinal lymphadenopathy and intra-abdominal lymphadenopathy. Cervical lymphadenopathy is not a common presentation of extrapulmonary sarcoidosis; however, a case of an isolated cervical lymph node sarcoidosis presenting with a neck mass has been reported in the literature [3]. The chest X-ray of this patient did not show mediastinal lymphadenopathy or reticular opacities as would be seen in pulmonary sarcoidosis. However, approximately 8% of patients with sarcoidosis present with disease at extrapulmonary sites without lung involvement [4]. Furthermore, a chest X-ray is a suboptimal test to screen for sarcoidosis in comparison to chest CT and MRI. Therefore, sarcoidosis should be considered a differential diagnosis in consideration of the possible variety in the presentation of extrapulmonary sarcoidosis and the low sensitivity of a chest X-ray to detect pulmonary sarcoidosis.

Lymphoma

Lymphomas can be divided into Hodgkin’s lymphoma and non-Hodgkin’s lymphoma. They both commonly present with lymphadenopathy and “B-symptoms,” which include fever, night sweats, and weight loss. Our patient presents with fever and lymphadenopathy of a prolonged duration unresponsive to antibiotics and conservative measures; therefore, lymphoma is considered as a differential diagnosis and should be ruled out with a biopsy due to its malignant nature.

Histiocytic Necrotizing Lymphadenitis (Kikuchi-Fujimoto Disease)

Kikuchi-Fujimoto disease or Kikuchi histiocytic necrotizing lymphadenitis is a benign condition originally described in young women. The most common clinical presentation of Kikuchi-Fujimoto disease is fever and cervical lymphadenopathy in previously well-young females and, therefore, should be considered in our patient. Lymph node biopsy is required for diagnosis despite the benign nature of the disease to exclude other serious causes such as neoplasia.

Castleman Disease (Angiofollicular Lymph Node Hyperplasia)

Castleman disease is a lymphoproliferative disease that can be classified into unicentric or multicentric disease based on the regions of involved lymph nodes. It commonly presents with lymphadenopathy, constitutional symptoms (fever, weight loss, and night sweats), hepatosplenomegaly, anemia, thrombocytopenia, elevated CRP/ESR, and hypergammaglobulinemia. A histopathological review of an excisional lymph node biopsy is required to diagnose Castleman disease. The clinical presentation is non-specific and is similar to our patient’s symptoms and findings; therefore, Castleman disease should be considered as a differential diagnosis in our patient.

Diagnostic tests

Lactate dehydrogenase level was elevated, measuring 700 IU/L. Beta-2 microglobulin was also elevated, measuring 3.02 mg/l. The kappa free light chain was above the normal level, measuring 26.4 mg/L. ACE level was normal. Repeated CBC showed microcytic anemia with hemoglobin of 97 g/L and leukopenia with neutropenia and lymphopenia measuring 1.54x10^9/L and 0.58x10^9/L, respectively. Iron studies revealed elevated ferritin, measuring 618 mcg/L, and transferrin below normal levels, measuring 1.80 g/L. C3 and C4 levels were within normal range, and anti-nuclear antibody and anti-double-stranded DNA were negative. HIV quantitative ​polymerase chain reaction (PCR) was negative. Mycobacterium TB PCR and Quantiferon-TB tests were both negative. Rapid plasma reagin was negative.

CT of the neck showed enhancing pathologically enlarged nodes seen in the neck bilaterally extending down to the base of the neck, associated with some soft tissue stranding particularly on the right side. In the chest, there are prominent variable-size lymph nodes noted in the right paratracheal, prevascular, and subcarina. In the abdomen, a CT image revealed pathological enhancement in enlarged lymph nodes along the right mesenteric vessels, with soft tissue stranding. A lymph node biopsy has been obtained.

Pathology discussion

H&E Stain

**Figure 1:**
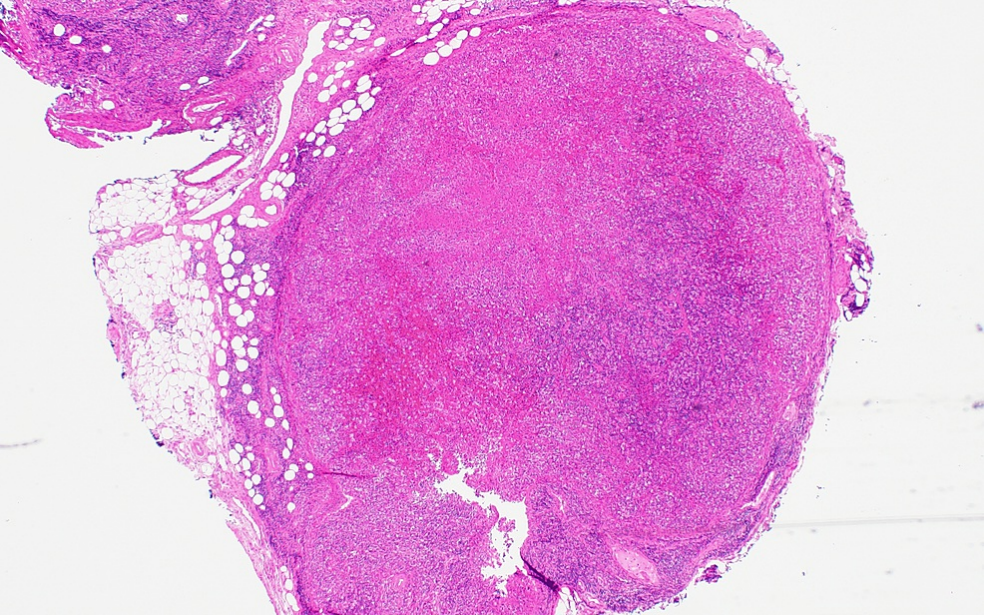
H&E stain sections showing mildly enlarged lymph node

**Figure 2:**
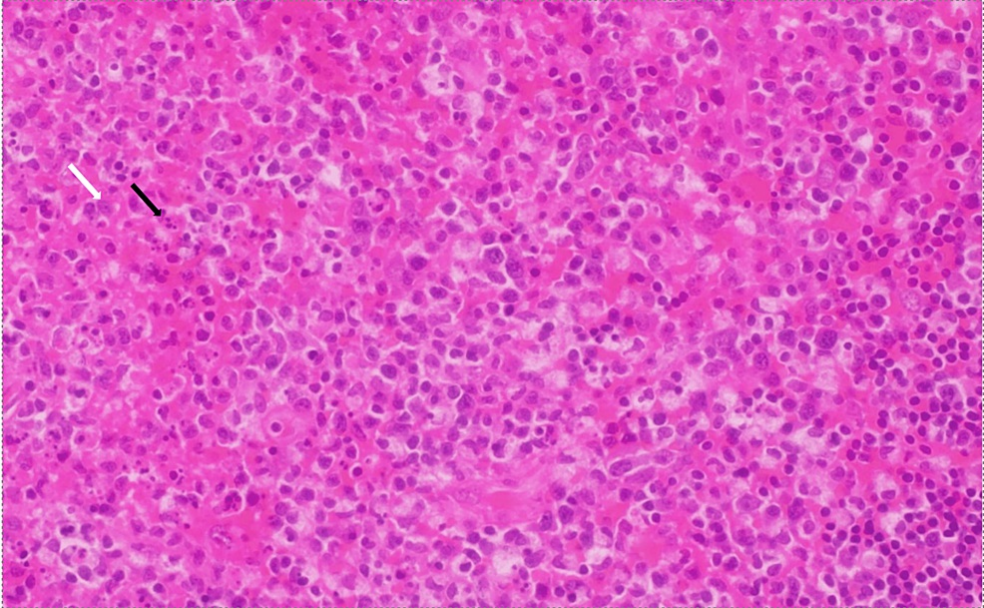
H&E stain section of a lymph node showing interfollicular/paratrabecular areas rich in histiocytes (white arrow) admixed with apoptotic bodies (black arrow)

Sections show mildly enlarged lymph nodes (Figure 1) with interfollicular/paratrabecular areas rich in histiocytes admixed with apoptotic bodies as seen (Figure 2). There is no prominence of neutrophils or eosinophils. No distinct Reed-Sternberg cells or other abnormal cells/cell clusters are evident.

Immunochemical Stain

CD21 and CD23 immunostains show focally retained follicular dendritic cell meshworks mostly at the edges of the specimen. CD3 and CD20/PAX5 immunostains show appropriate localization of the T cells and B cells to the interfollicular and follicular regions, respectively. The T cells co-express CD5 and BCL2. The B cells are negative for CD5, CD10, and cyclin D1. Focally, the general center B cells are highlighted by BCL6 with appropriate downregulation of BCL2. MUM1 highlights scattered interstitial plasma cells. Scattered CD30+ medium-sized cells are noted favored to be immunoblasts. No distinct HRS cells are evident by CD30, CD15, PAX5, and MUM1 immunostains. CD15 shows non-specific staining and is borderline suboptimal. The histiocytes are highlighted by CD68; histiocytes are also positive for MPO. The Ki-67 proliferation fraction is appropriately high in the germinal centers and low to moderate in the paracortical and interfollicular regions. All controls show appropriate reactivity. Flow cytometry analysis shows no overt immunophenotypic abnormalities in a partially vital sample.

The overall findings are consistent with Kikuchi lymphadenitis. Histologically, changes associated with autoimmune disease may mimic those seen in Kikuchi lymphadenitis; hence, clinical correlation, along with evaluation for possible autoimmune diseases, such as SLE, is recommended. There is no overt evidence of a hematolymphoid neoplasm in the submitted sample.

Final diagnosis

The final diagnosis is Kikuchi-Fujimoto disease (histiocytic necrotizing lymphadenitis).

Management

The patient was managed conservatively with antipyretics to control fever. She was scheduled for follow-up appointments at the internal medicine clinic to monitor symptoms. On subsequent follow-ups, the patient did not complain of fever or additional symptoms. She continues to follow up in the internal medicine clinic for recurrence or development of symptoms.

## Discussion

Kikuchi-Fujimoto disease was first reported in Japan in 1972 and has been reported in many countries since then. It usually affects young females, but it also occurs in males in a 4:1 ratio toward females [5]. Kikuchi-Fujimoto disease was reported in many different ethnic groups but mainly affects people of an Asian background. For instance, Song et al. found that around 34% of adult patients with cervical lymphadenopathy had Kikuchi-Fujimoto disease [6]. There is one case of Kikuchi-Fujimoto disease reported in the UAE by Shamsuldeen in 2016, where he reported Kikuchi-Fujimoto disease in a 27-year-old Southeast Asian female [7]. This case is the second case to be reported in an adult in the UAE.

The presentation of Kikuchi-Fujimoto disease is lymphadenopathy, mostly cervical. The involvement of extra-cervical lymph nodes is not common. Other symptoms include fevers, arthralgia, night sweats, arthritis, and rash [8]. Additional case reports reported other clinical presentations such as pleural effusion [9], hemolytic uremic syndrome (HUS) [10], and antiphospholipid syndrome with multiorgan failure [11]. Laboratory abnormalities vary between cases. Most patients with Kikuchi-Fujimoto disease have normal blood counts [5]. The analysis of 244 cases of Kikuchi-Fujimoto disease showed that around 43% of patients developed leukopenia. Anemia was reported in 23% of the patients [8]. Serologic studies such as antinuclear antibodies and rheumatoid factor are negative in most cases. However, these tests might be positive in patients with SLE who present with symptoms of Kikuchi-Fujimoto disease as their first presentation of SLE. There is an association with SLE described in the literature [12-14] as well as other diseases such as Still disease [15] and B cell lymphoma [16]. These findings suggest an accurate diagnosis should be made to ensure a proper follow-up for the patients. Some papers suggested a follow-up period of several years [5]. The gold standard for diagnosis is through a lymph node biopsy which shows nodules in the paracortex that consist of eosinophilic granular debris, histiocytes, plasmacytoid monocytes, and immunoblasts [17].

The treatment of Kikuchi-Fujimoto disease is mainly supportive. The disease usually self-resolves in one to four months [18]. Treatment usually involves analgesics and antipyretics. Although the prognosis is generally good, some patients might develop SLE in the future as previously discussed. Thus, a close follow-up for up to two years is recommended.

## Conclusions

In this paper, we reported a case of a 25-year-old female who presented with cervical lymphadenopathy and high-grade fever. Lymph node biopsy showed features suggestive of Kikuchi-Fujimoto disease. We believe that this is the second case reported in an adult in the UAE. Kikuchi-Fujimoto disease has a non-specific presentation that overlaps with a variety of diagnoses that encompass infectious, inflammatory, and malignant etiologies. Therefore, a thorough clinical approach is required. Kikuchi-Fujimoto disease is underdiagnosed due to the self-limiting nature of the disease. Although it is a benign disease, physicians should closely monitor their patients as some develop SLE, Still disease, or B cell lymphoma in the future.
